# Ecological dependencies and the illusion of cooperation in microbial communities

**DOI:** 10.1099/mic.0.001442

**Published:** 2024-02-22

**Authors:** Elze Hesse, Siobhán O’Brien

**Affiliations:** ^1^​ College of Life and Environmental Science, University of Exeter, Penryn, Cornwall, TR10 9FE, UK; ^2^​ Moyne Institute of Preventive Medicine, Department of Microbiology, School of Genetics and Microbiology, Trinity College Dublin, Dublin 2, Ireland

**Keywords:** cross-feeding, interdependencies, microbial communities, Public goods

## Abstract

Ecological dependencies – where organisms rely on other organisms for survival – are a ubiquitous feature of life on earth. Multicellular hosts rely on symbionts to provide essential vitamins and amino acids. Legume plants similarly rely on nitrogen-fixing rhizobia to convert atmospheric nitrogen to ammonia. In some cases, dependencies can arise via loss-of-function mutations that allow one partner to benefit from the actions of another. It is common in microbiology to label ecological dependencies between species as cooperation – making it necessary to invoke cooperation-specific frameworks to explain the phenomenon. However, in many cases, such traits are not (at least initially) cooperative, because they are not selected for because of the benefits they confer on a partner species. In contrast, dependencies in microbial communities may originate from fitness benefits gained from genomic-streamlining (i.e. Black Queen Dynamics). Here, we outline how the Black Queen Hypothesis predicts the formation of metabolic dependencies via loss-of-function mutations in microbial communities, without needing to invoke any cooperation-specific explanations. Furthermore we outline how the Black Queen Hypothesis can act as a blueprint for true cooperation as well as discuss key outstanding questions in the field. The nature of interactions in microbial communities can predict the ability of natural communities to withstand and recover from disturbances. Hence, it is vital to gain a deeper understanding of the factors driving these dynamic interactions over evolutionary time.

## Introduction

Ecological dependencies, where organisms rely on other organisms for survival, are a ubiquitous feature of communities across all domains of life. Humans rely on gut microbes to aid digestion of complex carbohydrates [1], and to synthesise essential vitamins [2, 3] and amino acids [4] for metabolism. Insects similarly host intracellular and extracellular symbionts that play beneficial roles in host nutrient provisioning, reproduction and defence [5]. The relationship between hosts and their associated microbiota is so intimately entwined, it has been controversially coined as the ‘holobiont’ – a synchronised unit on which natural selection could (possibly) act [6, 7].

Microbial communities themselves – host associated or not – arguably provide some of the best examples of fiercely interdependent communities. Microbial communities can contain potentially thousands of interacting species, wherein species may rely on other species for survival. For instance, B vitamins are synthesised by only 40–65 % of human gut microbial species. The remaining 35–60 % of our gut microbes lack B vitamin synthesis pathways – relying instead on vitamins synthesised by other bacterial residents of the gut [2]. Cross-feeding (the exchange of metabolites between bacteria and other micro- or macro-organisms [8]) has been reported between bacteria isolated from the human colon, oral cavity, soil and ocean ecosystems [8]. Samples from diverse environments show that the vast majority (potentially up to 99.9 %) of bacterial species cannot be cultured in the lab [9, 10] – but culturing techniques that grow cells in the presence of neighbouring microbes or even secreted products of neighbours can recover ‘unculturable’ bacteria [10] and increase the growth of auxotrophic mutants [11] (but see box 1). This paints a picture of a quintessential highly cooperative microbial community where species produce growth-essential factors and enzymes to benefit their host or neighbouring species. However, this viewpoint often misunderstands the driving force behind such dependencies, assigning loaded anthropogenic labels that makes the phenomenon difficult to explain. Here, we will describe a simple framework (coined as the ‘Black Queen’ [12, 13]) that explains the existence of some ecological dependencies observed in microbial communities, (usually) without needing to invoke cooperative labels that are often misapplied in studies of microbial interactions [14]. Due to the focus of this review, we concentrate on dependencies driven by gene-loss, where a loss-of-function mutation causes one species to become reliant on another species to fulfil some growth enhancing function. In contrast, gene-gain or replacement (arisen via mutations or horizontal gene transfer) can similarly drive dependencies if acquired traits allow species to utilize metabolites produced by neighbouring species. The latter is likely driven by resource competition and character displacement (divergence in resource use across species) and has already been discussed elsewhere, e.g. [8]. We also focus on ecological dependencies between microbes inhabiting the same trophic level since these are commonly confused with cooperation, but acknowledge that predator-prey and host-parasite interactions are also examples of dependencies in microbial communities.

Box 1. **How common are dependencies in natural microbial communities?**

The ‘great plate count anomaly’ – where the majority of species cannot be cultured under typical laboratory conditions – has been commonly cited as support for dependencies operating within natural microbial communities [10, 25]. If the vast majority of microorganisms are dependent on other species for growth, then this could logically explain why the vast majority of microbial species are unculturable in the lab. Computational analyses of metabolic networks supports this idea – the vast majority of bacterial species (free-living and endosymbionts) lack key genes for metabolic enzymes or pathways essential for metabolism [59]. Auxotrophic mutants are commonly isolated from natural communities, who are unable to provide all the nutrients they require for growth [79]. However, the conclusion that dependencies are so common that they can explain the prevalent unculturability of bacterial species has received some debate. Firstly, it is likely that our current culturing techniques are simply not advanced enough to capture all physiochemical components of relevant ecosystems [80]. Secondly, genomic analysis is shaped by our information, poor quality genomes, lack of annotations, and novel pathways can all potentially lead us to overestimate levels of dependency.

Efforts to quantify the extent of dependencies in natural microbial communities typically reveal that dependencies tend to be extremely rare [65, 66]. However, such findings have been criticised because (i) they (unavoidably) focus on culturable organisms only, which by definition are unlikely to be beneficiaries, and (ii) assays are performed under nutrient-rich conditions, where antagonistic interactions are predicted to prevail [31]. The debate goes on, but progress likely lies in combining culturing methods that more closely reflect natural conditions. Understanding the role dependencies play in natural microbial communities is a key part of predicting how microbial communities will respond to disturbances [70].

## The ‘problem’ of cooperation

**Fig. 1.**
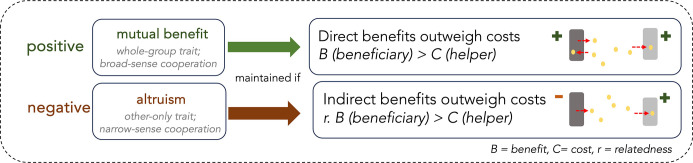
Cooperation (a trait that increases the fitness of a beneficiary and has evolved at least partly because of that benefit) can be divided into two categories depending on the net fitness effect carrying out the trait has on the helper [14]. Fitness here is measured as the effect of the trait on lifetime reproductive success (i.e. rate of cell division in bacterial populations). If performing the trait increases the fitness of the helper, the trait is mutually beneficial, since both parties benefit. Mutually beneficial traits can be selected for providing direct benefits to the helper outweigh any metabolic cost or trade-off associated with carrying out the trait (i.e*. B>C*). Mutually beneficial cooperation can occur between species, since high relatedness between partners is not required. Conversely, cooperation is altruistic when the helper pays a net fitness cost (i.e*. B<C*). In this case, cooperation can only be maintained if the helper gains indirect fitness benefits by increasing fitness of highly related individuals who are likely to share genetic material for the cooperative trait (i.e. *r. B>C*). The benefit to the beneficiary is weighted by average relatedness between interacting parties, so that only a small benefit to a highly related beneficiary is required to offset costs of carrying out the behaviour. The terms mutual benefit and altruism are also sometimes referred to as *(**i**)* broad or narrow-sense cooperation respectively, and *(ii)* whole-group or only-other traits respectively, given that the helper does not gain direct benefits in the latter.

The widespread occurrence of ecological dependencies in diverse ecosystems has been a key research question for microbiologists – particularly in the case of between-species dependencies observed in microbial communities [15–17]. Why should a microorganism help another unrelated species at an apparent cost to its own survival? How could this evolve? How would this be maintained in the long run, as organisms are expected to maximise their own fitness? These questions are more difficult to address if we consider the interaction through the lens of a cooperator/cheater prism, where a helper carries out a metabolically costly behaviour that benefits a recipient, and the behaviour is under selection (at least partly) because of those benefits to the recipient [14]. The maintenance of cooperation over evolutionary timescales relies on either direct benefits for the helper (i.e. both parties benefit from the interaction; mutual benefit; Fig. 1), or indirect benefits for the helper by increasing fitness of close relatives (i.e. the helper pays a cost while the recipient benefits; altruism; Fig. 1, box 2) [14]. Crucially, traits which carry a net cost for the helper will only be maintained when relatedness between interacting genotypes is high. In this way, a ‘gene’ for cooperation can be maintained in populations either by increasing the fitness of the helper directly, or indirectly by enhancing the fitness of other individuals who are also likely to harbour the same cooperative gene (i.e. kin selection). Note that the term ‘gene’ here is used conceptually, *sensu* [18] – in reality, cooperative traits likely require multiple genes for biosynthesis and transport of metabolites across cell envelopes [19].

Box 2. **Two criteria for cooperation**

Cooperation is defined here using the two criteria outlined by West *et al*. [14].

1. The trait must increase the fitness of the beneficiary
2. The trait must be selected for due to the beneficial effects on the beneficiary

Note, we use the term ‘beneficiary’ analogously to ‘recipient’ used by West *et al.*

In microbiology, interactions are often labelled as cooperative when the presence of one species facilitates the growth of another [2, 20, 21]. However, while this fits the first criteria for cooperation (recipient gains a fitness benefit), it does not always fit the second criteria – that the trait is at least partly selected due to fitness effects on the beneficiary [14]. The latter criteria is typically absent from reports illustrating interspecies ‘cooperation’, but it is important for distinguishing cooperation from simple by-product facilitation. A nice analogy, described by West *et al.* 2007 [14] is the effect of elephant dung on the activity of a dung beetle. The trait (producing dung) benefits the recipient (dung beetle) but the elephant is clearly not cooperating with the dung beetle because the act of producing dung is not selected for because of its effect on dung beetles. Similarly, the production of waste products or enzymes by one bacterial species might benefit a second species; the recipient benefits (just like the dung beetle), but waste production is not under selection because of its effect on a second species. Such interactions can eventually transform into cooperation (discussed below), but at least at the outset, there is no need to invoke cooperation-specific explanations for the behaviour. This over-tendency to label positive interactions as cooperation leads to problems explaining the behaviour, because true cooperation can only exist under a defined set of conditions (Fig. 1, Table 1).

**Table 1.** Assumptions of cooperation versus Black Queen frameworks

Cooperation	Black Queen dependencies
Requires high relatedness between interacting partners in absence of direct benefits for helper.	Reduces assumptions about costs and relatedness between interacting parties.
Trait must be metabolically costly to produce by both helper and beneficiary.	Trait may or may not be costly for helper, but is always costly for a potential beneficiary prior to loss-of-function mutation.
Trait must be under selection at least partly because of fitness benefits accrued by beneficiary.	No requirement for trait to be under selection due to fitness benefits accrued by beneficiary.
Cooperation between species requires restrictive ecological conditions.	Dependencies between species can arise through simple loss-of-function mutations.
Can be applied to narrow range of traits, such as public goods.	Can be applied to broad classes of traits, including leaky metabolites, extracellular public products, and intracellular detoxification.

## Enter the Black Queen

The ‘problem’ of interdependencies could also be viewed through the lens of the Black Queen (BQ) – a framework that describes the emergence of dependencies in microbial communities as a result of reductive evolution and loss of metabolic functions [12, 13]. There are no cooperators here – only passive helpers – who increase the growth of other species inadvertently. The hypothesis considers a BQ function as a microbial trait that is both *beneficial* and *leaky* (partially available to the broader community). Production of the traits by a recipient cell must also carry a *metabolic cost*, which can be ameliorated via a loss-of-function mutation. The BQ hypothesis predicts that simple one-step inactivating mutations will produce non-producing mutants that can coexist with producing strains due to simple negative frequency dependence [12]. In other words, the helper becomes ‘stuck’ carrying out some costly behaviour and trapped in its role as a helper. This is distinct from the view that helpers are altruists or cooperators that act to benefit nearby cells, and because it does not invoke cooperation, there is no requirement for high relatedness between interacting cells. True cooperative traits (Fig. 1) can indeed form a subset of BQ traits (for example, some public goods), but not all BQ traits are cooperative. Below, we summarise three categories of potentially leaky BQ functions described by Morris *et al.* [12] and give concrete examples of how the BQ framework can predict the emergence of dependencies in microbial communities.

### Release of leaky metabolites

**Fig. 2.**
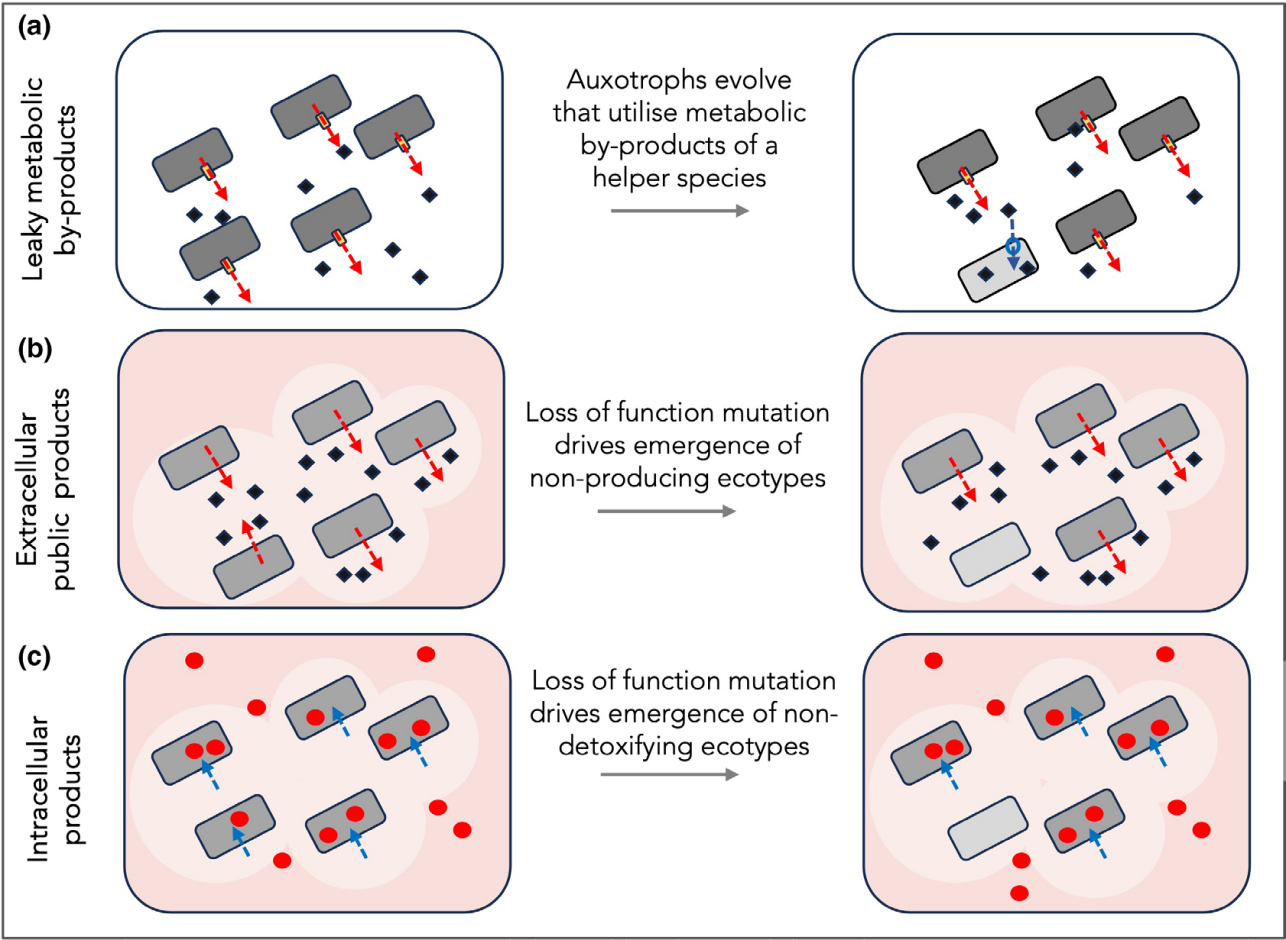
Three categories of leaky BQ functions. (**a**) Metabolic by-products or waste products are exported from cells (red arrows) to avoid build-up of toxic levels of metabolites in cells. Novel ecotypes may evolve *de novo* (light grey cells) that lose the ability to synthesise the corresponding metabolite, instead relying on beneficiaries to produce the metabolite. This leads to a dependancy, where the presence of dark grey cells increases the growth of light grey cells. (**b**) Many extracellular products can benefit nearby cells, for example by detoxifying the local environment. Areas of lighter coloured circles indicate detoxified regions where a non-detoxifying ecotype (light grey) may be protected. (**c**) The sequestration of toxic compounds intracellularly can create pockets of less-toxic space where non-detoxifying ecotypes can survive. In each of the cases a–c, a simple loss of function mutation can lead to the emergence of a dependency, where one species or population can facilitate the growth of another. Cells appear as grey rods in this figure for simplicity, but interdependencies can be formed between populations of the same or different species, as there is no requirement for high relatedness between partners. Blue diamonds represent a BQ product released extracellularly (**a and b**), and red circles indicate a toxin that is degraded intracellularly by helper bacteria.

Bacterial metabolism is an imperfect process, where overproduced metabolic intermediaries and waste-products must be degraded or removed from cells to avoid toxic levels of accumulation. As many bacteria lack the degradation machinery to cope with metabolic by-products intracellularly, the products must either leak or be actively transported from cells using efflux systems (relief valves; [22, 23]). Cell lysis can similarly result in the release of proteins, lipids, sugars, and nucleic acids into the environment [24]. Such leaky by-products can pave the way for the emergence of an auxotrophic consumer that can specialise on these leaky products (i.e. one-way by-product cross-feeding [22–25], Fig. 2a). This prediction was verified using experimental evolution of *Escherichia coli* in environments containing amino acids. In fewer than 2000 generations, auxotrophic genotypes evolved *de novo* that failed to synthesise their own amino acids – instead acquiring amino acids from the environment or by cross-feeding from amino acid synthesising strains [26].

Cross-feeding interactions can occur within or between species, but sustained supply of the metabolic by-product, and co-localization of the two subtypes must be maintained to allow auxotrophs to become fixed in the population. The trait is *beneficial* for both parties (although the benefits CAN take different forms – avoiding toxic accumulation in the helper and provision of important nutrients in beneficiary). The trait is also *costly to produce* independently by the future beneficiary. Such cross-feeding interactions give the illusion of complex interspecies cooperation, yet can simply be leaky products that benefit other cells as a ‘*necessary by-product of doing business’* [13]*.*

Bacterial metabolism is not perfect, but it can become even more wasteful in stressful or fluctuating environments. Physiochemical stressors can upregulate stress response systems that increase membrane permeability and release essential metabolites from cells [27]. Environmental fluctuations can cause a mismatch between a cell’s metabolic network architecture and the external environment [28]. For instance, if amino acids present in the environment are unbalanced with cell metabolic architecture, surplus amino acid will be excreted [29]. This indicates that environmental stress should result in more positive interactions between members of a microbial community, because there is more scope for cross-feeding of leaky metabolites. Hence, environmental stress will impact the structure of microbial communities, creating ecological opportunities for positive, cross feeding interactions. Empirical evidence supports the idea that the frequency of positive social interactions increases when natural microbial communities are subjected to environmental stress [30, 31].

### Extracellular public products

Bacteria possess a diverse repertoire of secreted proteins and metabolites, many of which allow the establishment of a favourable microenvironment. Secreted proteins function in defence against predators, host immune cells and competing bacteria, as well as in nutrient scavenging and toxin degradation [32–34]. Extracellular secretions can allow the re-design of an unfavourable microenvironment to resemble conditions to which a pathogen is most closely adapted [35]. In this way, possessing a diverse repertoire of extracellular secretions is a good indication of whether a pathogen is likely to be restricted to a specific host species, or to become a generalist pathogen that can infect multiple host species [35]. Secreted proteins are therefore important for protecting against hostile conditions, potentially creating time and space for *de novo* adaptation to a novel environment. Indeed, many secreted metabolites lie on mobile genetic elements, potentially allow multiple species in a community to rapidly adapt to suboptimal conditions [36] (but see [37]). Secreted proteins are *leaky* – that is, secretion of an environment-modifying protein can also create a favourable habitat for other strains and species [38] (Fig. 2b). Secreted proteins are also *costly for helpers to produce* (in contrast with leaky BQ functions mentioned above, which are only costly for the potential beneficiary to produce), yet they can carry direct or indirect benefits for the helper that ensure the fitness benefits outweigh the costs. A canonical example is the formation of biofilms that protect cells from external stressors, such as antibiotics, innate immune cell attack and detergents [39]. In *P. aeruginosa*, biofilm forming phenotypes secrete extracellular polysaccharides that form the scaffold matrix of the biofilm [40]. Non-producing phenotypes also live in the biofilm, but they avoid costs of producing polysaccharides, instead relying on helpers to maintain the biofilm structure [41]. Similarly, in a four-species community of soil isolates, one species *– Xanthomonas retroflexus –* formed biofilms that could support the growth of the three other species, despite the fact that the three remaining species couldn’t form a biofilm in isolation [42]. In the four-species biofilm, both biofilm biomass and the abundance of all four species was higher in a consortia than when growing as single-species biofilms, provide direct advantages for all species. Siderophores and other extracellular metabolites can release bioavailable nutrients, usable by other strains and species as well as the producer [43]. Enzymes that degrade toxins extracellularly (e.g. some metal and antibiotic degradation enzymes) can also benefit nearby cells [30, 44–46]. The secretion of toxins can similarly carry leaky benefits, if killing competing species or evading host immunity provides advantages for neighbouring bacteria [47].

In some cases, extracellular products are canonical examples of public goods – that is, they are under selection because of their beneficial effects on close relatives and group-level benefits [48]. Sharing of public goods is an example of mutual benefit cooperation (Box 2), and selection on such traits likely operates within-species, where indirect benefits and collective benefits will matter. A variety of microbial traits have been proposed as potential public goods, including extracellular proteases, iron-scavenging siderophores, group motility, toxin production and biofilm formation [48]. While selection for public goods production may operate primarily within-species, it may be inevitable that such traits may inadvertently benefit unrelated species, creating scope for BQ dynamics between species. This begs the question of whether such accidental facilitation opens up opportunities for between-species exploitation (+/−), or if the interaction will remain as facilitation (+/0). The answer likely depends on who receives the majority of the benefits – if benefits are largely directed to kin than a second species may not fully exploit the producer, and the interactions between species will be commensal (rather than exploitative). This is somewhat analogous to findings from metal polluted compost communities. Copper-sensitive species benefit from siderophores/copper-detoxifying compounds produced by another species, but this could not fully rescue the former’s growth, such that it did not lead to exploitation of the producer [49]. These dynamics reveal the complex patterns of selection that may operate in microbial communities, leading to multiple, contrasting forms of dependencies both within and between species.

#### Intracellular detoxification

Intracellular processes can also be leaky when they alter the extracellular environment. Some marine organisms can detoxify HOOH via catalase-peroxidase (katG), creating a HOOH sink and reducing HOOH in the environment. Some oxygen-producing species (e.g. *Prochlorococcus*) lack detoxication genes, relying on katG activity of helper strains [50]. Some toxic metals can similarly be compartmentalised in the periplasm of bacterial cells [51] or degraded to a less-toxic form prior to efflux [52], reducing environmental toxicity. Intracellular detoxification is leaky (since it could benefit nearby cells, as well as the producer) and essential (to avoid toxic build up in cells), and so could be regarded as a BQ function [53], Fig. 2c.

For example, the copABCD operon in the plant pathogen *Pseudomonas syringae* facilitates periplasmic accumulation of copper [54]. While copper accumulation carries direct benefits, it may also benefit neighbouring cells by reducing the net concentration of copper in the environment. Samples from commercial mango orchids in Portugal exposed to copper-containing bactericides found resistance to cupric sulphate in ~59 % of *P. syringae* isolates [54], providing ample scope for BQ dynamics. Antibiotics can similarly be degraded intracellularly, for example via intracellular β-lactamases or acetyl transferases. Some strains of *Streptococcus pneumoniae* (causative agent of pneumococcal disease) express chloramphenicol acetyltransferase (CAT), conferring resistance to chloramphenicol. CAT deactivates chloramphenicol intracellularly, providing direct benefits for CAT-expressing cells but incidentally reducing chloramphenicol concentrations in the environment. In a mouse pneumonia model, Sorg *et al.* [55] reported that while sensitive cells are inhibited by chloramphenicol in isolation, they can survive and colonize the host in the presence of CAT-expressing cells.

While such studies are typically contrived (a susceptible strain is introduced rather than evolving *de novo*), they predict BQ dynamics at least have the potential to arise from intracellular detoxification traits. However, it is still unclear how BQ dynamics may shape intracellular detoxification, especially in mixed-species microbial communities.

## Loss of function mutations as drivers of dependencies

For each of these BQ functions, simple one-step inactivating mutations can produce mutants that no longer perform the function (beneficiaries), and are able to invade and stably coexist with others who perform the function (helpers) [12, 13]. Loss of a beneficial metabolic trait can be due to genetic drift/small population sizes (particularly common in endosymbionts [56]), or by natural selection favouring resource conservation (more common in free living organisms) [57]. Note that there are alternative explanations for trait loss, such as the trait no longer being required, but such processes would not create beneficiaries. Beneficiaries gain a fitness benefit via gene deletion, not from genomic streamlining per se, but because energy is conserved by eliminating protein biosynthesis [58, 59]. Notably, at this point, these relationships are not necessarily cooperative (box 2). The helper is simply the unfortunate player who ended up stuck in that role, and the beneficiary is the lucky one who won the game.

This process is repeated for multiple leaky traits across multiple species, so communities then consist of function-performing helpers and function-depending beneficiaries. This partitioning of benefits can cause negative frequency dependence in microbial communities – allowing coexistence between multiple ecotypes in a community. Frequency dependence has similarly been invoked to explain the evolution of stable polymorphisms in bacterial populations [60]. The ubiquity of leakiness facilitates a ‘race to the bottom’ as members of a community lose the ability to perform functions whose products are available from the environment. Rather than being cooperators or altruists, these helpers are merely populations that lost this race and got stuck in their role as function performers.

## Predicting roles: who will play the Black Queen?

The BQ hypothesis provides a useful framework for thinking about how interdependencies formed from leaky traits give the illusion of cooperation in microbial communities. However, one outstanding question is whether simple rules can predict which species will evolve to become beneficiaries, and which species will become confined to their role as helper. Species with higher mutation rates or larger population sizes may be more likely to end up as beneficiaries, as the chance of acquiring a loss-of-function mutation is higher. Differential opportunity costs among species in a community could also determine differentiation into helpers/beneficiaries. Adkins-Jablonsky *et al.* [61] tested this idea using two distinct ecotypes of *E. coli* that were genetically modified to live on mutually exclusive resources (mannose and galactose) and hence occupied separate niches. Both ecotypes contained an ampicillin-resistance plasmid (pBQ1) containing genes encoding the enzyme β-lactamase for degrading ampicillin extracellularly (i.e. a BQ function). The team hypothesised that evolution should drive a race to the bottom, where one ecotype gets trapped as the ampicillin-degrading helper, while the second ecotype should become the antibiotic-susceptible beneficiary. The question was, whether each ecotype will end up in the helping role approximately 50 % of the time (i.e. random allocation), or whether one ecotype predictably ends up in the role of helper. They found the latter – in 15/18 replicate evolving populations, galactose metabolisers became susceptible to ampicillin, relying instead on ampicillin degradation by mannose-metabolising helpers, who retained antibiotic resistance throughout the experiment. This was explained by mannose metabolisers having a lower intrinsic level of ampicillin resistance, so that the benefit of expressing the resistance gene was higher. Hence, the higher opportunity cost experienced by one ecotype could predict which ecotype specialised into which role, despite strains being genetically identical apart from their niche-defining mutation. This idea of ‘silently advantageous’ traits held by future beneficiaries was also demonstrated theoretically using agent based modelling [62]. It is unclear whether this prediction would scale up to natural microbial communities, where mutation rates and genomic constraints will differ between and within species.

It is worth noting at this point that even when partners are reliably encountered, restrictive ecological conditions are required to support the maintenance of dependencies between different strains or species. Firstly, ecological theory predicts that niche overlap between different genotypes will result in exploitative competition, with the intensity of resource competition proportional to the degree of niche overlap [63]. However, in some circumstances, interacting partners can occupy distinct niches, as was the case in Adkins-Jablonsky *et al*. [61] study discussed above, where *E. coli* ecotypes either specialised on mannose or galactose. Since there is little merit in helping a competitor, positive interactions between genotypes will only be maintained when resource overlap between interacting partners is low [17]. Secondly, costly BQ functions (such as degradation enzymes or nutrient scavengers) may take the form of true cooperation within a species, even though the benefits they confer to other species are incidental, rather than cooperation. For example, the production of metal-chelating siderophores is likely a form of cooperation within a species, since fitness benefits accrued by clonemates at least partly drive selection for production [64]. In such cases, high within-species relatedness is required to sustain the production of a costly secretion within that species [17]. The restrictive criteria that must be met to maintain positive interactions between different genotypes aligns with findings that microbial communities are dominated by negative, rather than positive interactions [65, 66]. However, it is clear that restricting analyses to culturable microbes in a single environment will underestimate the frequency of interdependencies in natural microbial communities, since culturable bacteria, by definition, can grow in the absence of neighbouring species [67]. Furthermore, there is a lower probability of observing positive interactions in nutrient-rich conditions (typically employed in such assays), where theory predicts competition to prevail [68].

## Black queen interdependencies as a blueprint for cooperation

**Fig. 3.**
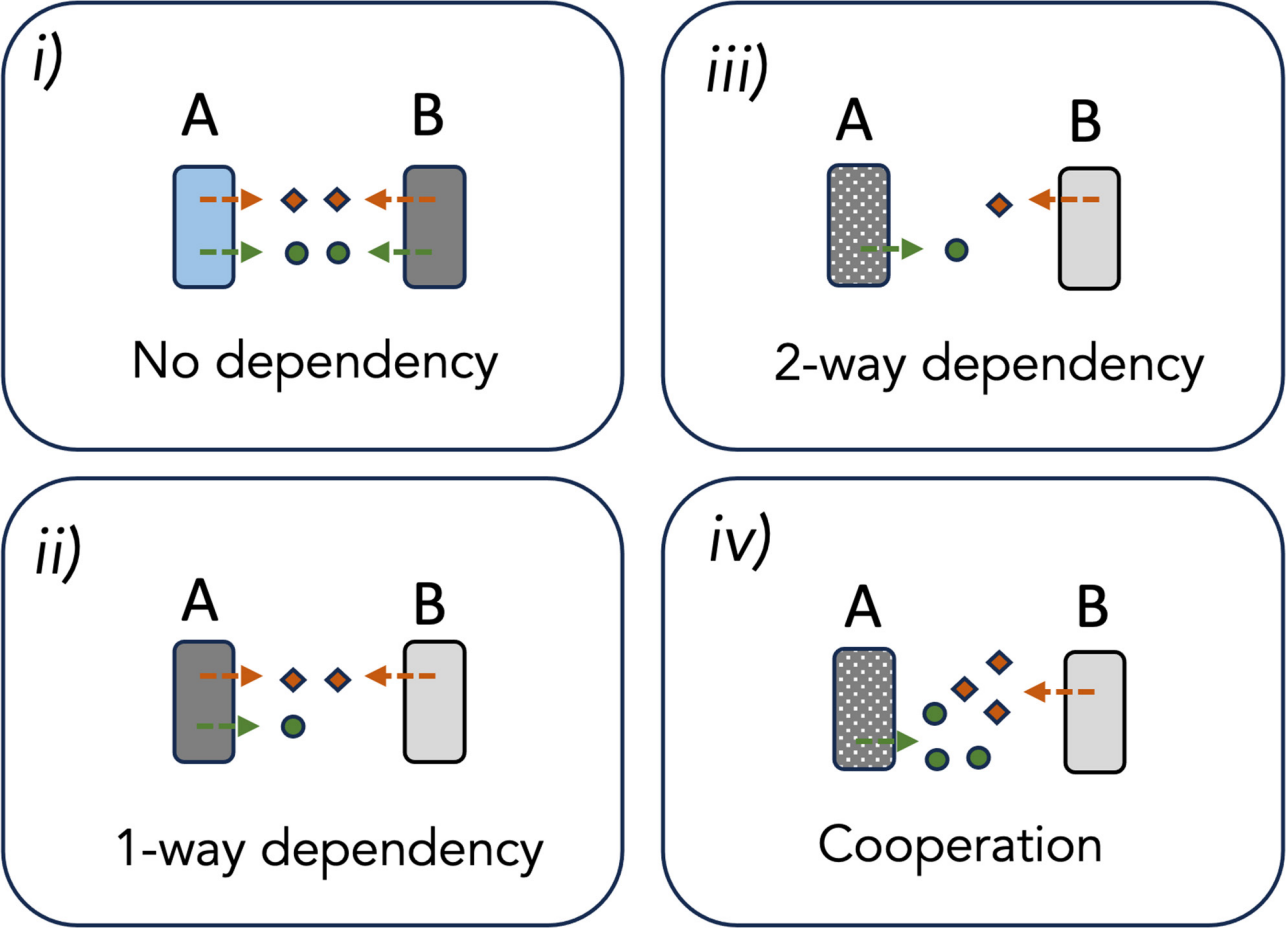
Stages of forming a cooperative mutualism via loss of function mutations and BQ dynamics. **i**) Two ecotypes (A and B) secrete two distinct BQ functions into the extracellular environment (i.e. orange diamonds and green circles). The functions carry direct benefits for the producing cells and are associated with enhanced growth of the producer (e.g. detoxifying enzymes). ii) Ecotype B evolves a loss-of-function mutation for the green BQ function, making it a beneficiary of ecotype A for the green trait. The interaction is now a one-way form of facilitation. iii) A reciprocal loss of function mutation occurs in ecotype A, this time in the orange BQ function, making it a beneficiary of ecotype B for the orange trait (two-way facilitation). iv) Selection may act on both traits due to the growth-stimulating effect on the partner species. The interaction is now truly cooperative, because each ecotype is producing more than they actually need to sustain growth, i.e. the traits are under selection due to the benefits they confer on beneficiaries.

The BQ framework shows how interdependencies form in communities as a result of leaky traits. However, under certain conditions, selection can act on these interdependencies to form true cooperative interactions. If helpers evolve mutations that force beneficiaries to ‘*earn their keep’* [12], then BQ evolution can act as a catalyst for generating mutually beneficial cooperation. For instance, if a helper evolved a loss of function mutation that made it dependant on a leaky BQ function secreted by the beneficiary (e.g. two-way cross-feeding) then conditions required to transition to a mutualistic form of cooperation may be met. However, to satisfy criteria for cooperation, selection must act on leaky traits via fitness effects on both parties, so each partner is producing more of a given metabolite than they actually need to sustain their own growth. For example, if species A increases production of a leaky BQ function, it gains direct fitness benefits by enhancing the growth of species B and hence access to the beneficial leaky function secreted by species B. Species B may similarly increase the production of some leaky BQ function, benefitting directly via enhanced growth of species A on which they rely [8]. At this point a mutualistic form of cooperation is established, because both parties benefit, and selection acts on traits at least partly due to fitness effects on beneficiaries (Fig. 3). However, it is difficult to test whether an interaction between two natural isolates is truly cooperative, because isolates not engaging in the behaviour are needed as a baseline against which the focal isolates are compared [8].

## Future research

**Fig. 4.**
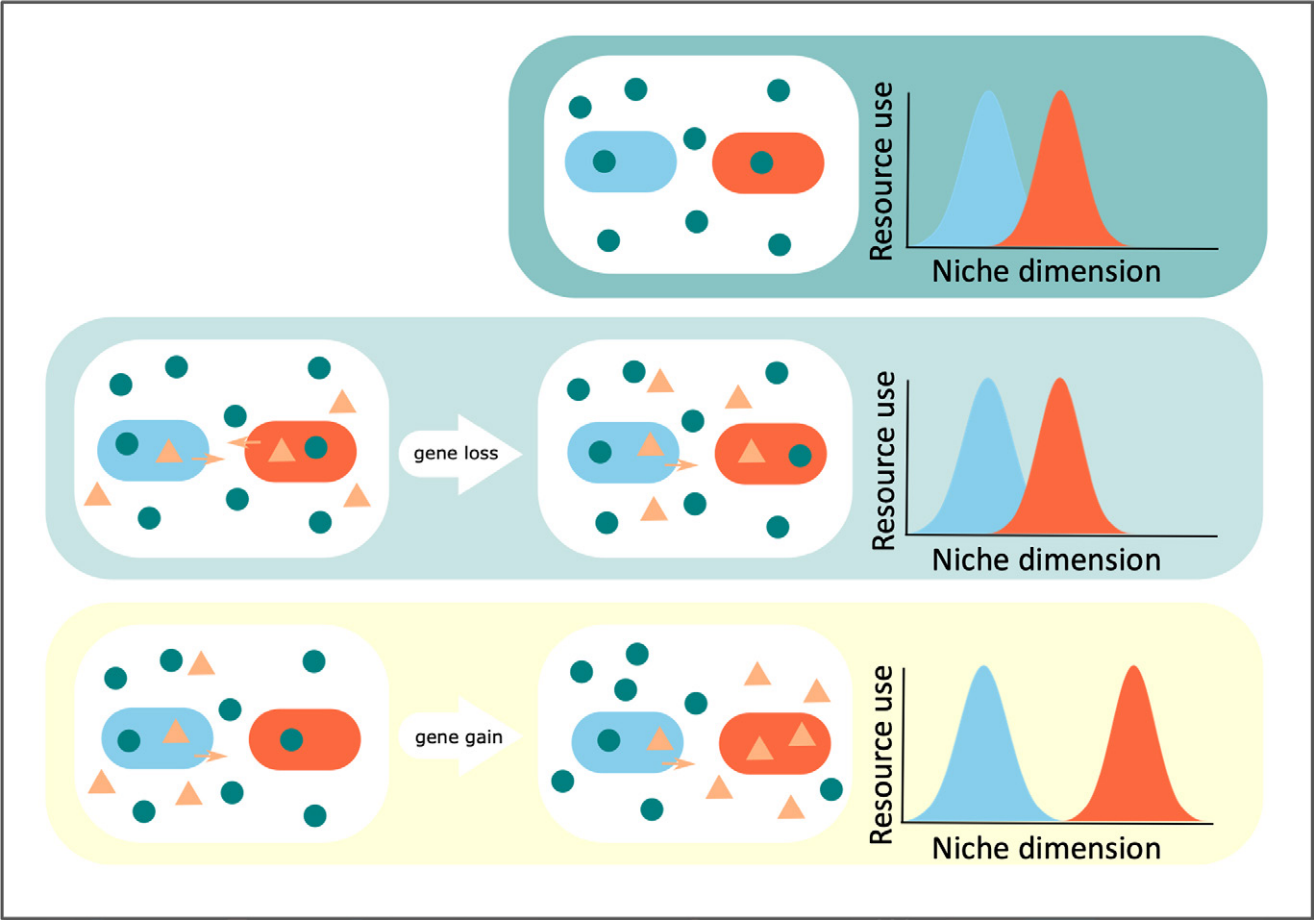
The consequences of reductive evolution and gene gain on resource partitioning in ecological dependencies. Top panel: Species (red and blue) often overlap in resource use as a result of habitat filtering, meaning they often engage in competition for limiting resources (green circles). Middle panel: When these species release products into the environment that benefit others (orange triangles), then this might result in the emergence of a loss-of-function mutation in one of the species (orange species), which then relies on the blue species to obtain this 'leaky' good. In this scenario, both species still compete for the same limiting resources, and as such, the qualitative nature of the interaction does not change (remains negative). In the extreme, if the loss of function mutation frees up resources to obtain limiting resources then the orange species could eventually outcompete the blue species (as the latter bears the cost of producing the leaky good). Bottom panel: The blue species releases a metabolite (by-product or waste) that is not of benefit to itself. Resource competition between species could select for specialisation on this metabolite by the orange species. This reduces resource overlap and hence ameliorates competition between the two species.

Most evidence for BQ dynamics comes from retrospective studies of genome sequences (reviewed in [62]). Because these studies are inherently correlational in nature, it remains unclear whether apparent cross-feeding dependencies have emerged from reductive evolution (shared BQ functions) or gain of function mutations (niche partitioning and resource divergence) over time. It is important to distinguish between these inherently different evolutionary processes as they are likely to have different consequences for community functioning. Given that co-occurring species often display a degree of resource overlap, gene loss dependencies are more likely to be antagonistic compared to gene gain dependencies, especially if the latter results in the emergence of complementary resource use among competing species (e.g. when a species evolves a novel ability to use another species’ waste, and thereby reduces interspecific competition [69]; Fig. 4). If the beneficiary species has a net positive effect on the helper, for example, by metabolizing toxic waste, then such dependencies could eventually become mutually beneficial, potentially leading to a division of labour. Theory predicts that the degree of antagonistic and mutualistic interactions in microbial communities greatly affects the productivity and stability of microbial communities [70].

Because microbial communities are typically comprised of hundreds of species (or more), it is challenging to study evolution in the wild. In recent years, researchers have started using experimental evolution to track evolutionary changes in increasingly complex communities (e.g. [31, 71–73]). However, experimental studies investigating BQ dynamics are rare and have mainly used closely related strains to demonstrate BQ dynamics [26, 74]. It is unclear whether these results can be translated to more complex environments, where opportunities for sharing of leaky goods fluctuate over space, time and across species. For example, the production of leaky goods often benefit related individuals as well as other community members. Examples of such leaky goods include antibiotic-degrading enzymes [75, 76], immune-manipulating effectors [77], and resource-scavenging molecules [64]. Selection pressures acting on these leaky goods are inherently different from those at play in dependencies based on leaky traits that have differential functional roles in the helper versus beneficiary species. What happens when leaky goods can be exploited/used by individuals of both the same and different species? Will their dynamics be largely determined by kin selection or by direct costs and benefits associated with the sharing of leaky goods? If the latter is the case, can this result in a division of labour, where each of the interacting species specializes to carry out a certain function?

Another knowledge gap concerns the occurrence of BQ dependencies across different environments. Most studies demonstrating BQ dynamics in free-living bacteria have done so in aquatic – often nutrient-poor – environments. Under these conditions it may pay off to economise resource use, and rely on others to carry out costly functions if these are publicly available. It is unclear to what extent nutrient availability affects BQ dynamics in a community context (but see [26] for emergence of cross-feeding in *E. coli*), and whether loss-of-function mutants readily evolve in nutrient-replete environments under more natural conditions (e.g. soil). For example, if the cost of carrying out a leaky function is reduced when nutrients are relatively abundant, but that function is still essential for growth, then selection favouring gene loss might be relaxed (analogous to reduced incentives of ‘cheating’ in public goods dynamics [78]). Alternatively, a loss-of-function mutation might arise when metabolites can be derived from the environment, or when the lost function is not required for survival. These opposing selection pressures might cancel each other out, depending on the complexity of the environment and that of the community. A further complicating factor is the spatial of heterogeneity in natural systems, which is often overlooked in experimental studies of BQ dynamics. This raises the question as to how spatial structure affects the emergence and maintenance of dependencies, both within and between species?

To our knowledge, the above questions have not been experimentally tested thus far. There is clear need for more experimental studies that directly measure and manipulate conditions that favour gene loss and the evolution of dependencies in a community context.

## References

[1] Martens EC, Lowe EC, Chiang H, Pudlo NA, Wu M (2011). Recognition and degradation of plant cell wall polysaccharides by two human gut symbionts. PLoS Biol.

[2] Magnúsdóttir S, Ravcheev D, de Crécy-Lagard V, Thiele I (2015). Systematic genome assessment of B-vitamin biosynthesis suggests co-operation among gut microbes. Front Genet.

[3] LeBlanc JG, Milani C, de Giori GS, Sesma F, van Sinderen D (2013). Bacteria as vitamin suppliers to their host: a gut microbiota perspective. Curr Opin Biotechnol.

[4] Lin R, Liu W, Piao M, Zhu H (2017). A review of the relationship between the gut microbiota and amino acid metabolism. Amino Acids.

[5] Salem H, Kaltenpoth M (2022). Beetle-bacterial symbioses: endless forms most functional. Annu Rev Entomol.

[6] Rosenberg E, Zilber-Rosenberg I (2018). The hologenome concept of evolution after 10 years. Microbiome.

[7] Douglas AE, Werren JH (2016). Holes in the hologenome: why host-microbe symbioses are not holobionts. mBio.

[8] D’Souza G, Shitut S, Preussger D, Yousif G, Waschina S (2018). Ecology and evolution of metabolic cross-feeding interactions in bacteria. Nat Prod Rep.

[9] Wade W (2002). Unculturable bacteria--the uncharacterized organisms that cause oral infections. J R Soc Med.

[10] D’Onofrio A, Crawford JM, Stewart EJ, Witt K, Gavrish E (2010). Siderophores from neighboring organisms promote the growth of uncultured bacteria. Chem Biol.

[11] Kost C, Patil KR, Friedman J, Garcia SL, Ralser M (2023). Metabolic exchanges are ubiquitous in natural microbial communities. Nat Microbiol.

[12] Morris JJ (2015). Black Queen evolution: the role of leakiness in structuring microbial communities. Trends Genet.

[13] Morris JJ, Lenski RE, Zinser ER (2012). The Black Queen hypothesis: evolution of dependencies through adaptive gene loss. mBio.

[14] West SA, Griffin AS, Gardner A (2007). Social semantics: altruism, cooperation, mutualism, strong reciprocity and group selection. J Evol Biol.

[15] Mitri S, Xavier JB, Foster KR (2011). Social evolution in multispecies biofilms. Proc Natl Acad Sci U S A.

[16] Oliveira NM, Niehus R, Foster KR (2014). Evolutionary limits to cooperation in microbial communities. Proc Natl Acad Sci U S A.

[17] Mitri S, Foster KR (2013). The genotypic view of social interactions in microbial communities. Annu Rev Genet.

[18] Dawkins R (1976). The Selfish Gene.

[19] Waters CM, Bassler BL (2005). Quorum sensing: cell-to-cell communication in bacteria. Annu Rev Cell Dev Biol.

[20] Cockburn DW, Koropatkin NM (2016). Polysaccharide degradation by the intestinal microbiota and its influence on human health and disease. J Mol Biol.

[21] Adak A, Khan MR (2019). An insight into gut microbiota and its functionalities. Cell Mol Life Sci.

[22] Gude S, Pherribo GJ, Taga ME (2020). Emergence of metabolite provisioning as a by-product of evolved biological functions. mSystems.

[23] McKinlay JB (2023). Are bacteria leaky? Mechanisms of metabolite externalization in bacterial cross-feeding. Annu Rev Microbiol.

[24] Pherribo GJ, Taga ME (2023). Bacteriophage-mediated lysis supports robust growth of amino acid auxotrophs. ISME J.

[25] Pande S, Kost C (2017). Bacterial unculturability and the formation of intercellular metabolic networks. Trends Microbiol.

[26] D’Souza G, Kost C (2016). Experimental evolution of metabolic dependency in bacteria. PLoS Genet.

[27] Rhodius VA, Suh WC, Nonaka G, West J, Gross CA (2006). Conserved and variable functions of the sigmaE stress response in related genomes. PLoS Biol.

[28] Waschina S, D’Souza G, Kost C, Kaleta C (2016). Metabolic network architecture and carbon source determine metabolite production costs. FEBS J.

[29] Lopez JG, Wingreen NS (2022). Noisy metabolism can promote microbial cross-feeding. Elife.

[30] Hesse E, O’Brien S, Luján AM, Sanders D, Bayer F (2021). Stress causes interspecific facilitation within a compost community. Ecol Lett.

[31] Piccardi P, Vessman B, Mitri S (2019). Toxicity drives facilitation between 4 bacterial species. Proc Natl Acad Sci U S A.

[32] Lee VT, Schneewind O (2001). Protein secretion and the pathogenesis of bacterial infections. Genes Dev.

[33] Ma W, Guttman DS (2008). Evolution of prokaryotic and eukaryotic virulence effectors. Curr Opin Plant Biol.

[34] Bassler BL, Losick R (2006). Bacterially speaking. Cell.

[35] McNally L, Viana M, Brown SP (2014). Cooperative secretions facilitate host range expansion in bacteria. Nat Commun.

[36] Rankin DJ, Rocha EPC, Brown SP (2011). What traits are carried on mobile genetic elements, and why?. Heredity (Edinb).

[37] Hao C, Dewar AE, West SA, Ghoul M (2022). Gene transferability and sociality do not correlate with gene connectivity. Proc Biol Sci.

[38] Garcia-Garcera M, Rocha EPC (2020). Community diversity and habitat structure shape the repertoire of extracellular proteins in bacteria. Nat Commun.

[39] Costerton JW, Lewandowski Z, Caldwell DE, Korber DR, Lappin-Scott HM (1995). Microbial biofilms. Annu Rev Microbiol.

[40] Ryder C, Byrd M, Wozniak DJ (2007). Role of polysaccharides in *Pseudomonas aeruginosa* biofilm development. Curr Opin Microbiol.

[41] Popat R, Crusz SA, Messina M, Williams P, West SA (2012). Quorum-sensing and cheating in bacterial biofilms. Proc Biol Sci.

[42] Ren D, Madsen JS, Sørensen SJ, Burmølle M (2015). High prevalence of biofilm synergy among bacterial soil isolates in cocultures indicates bacterial interspecific cooperation. ISME J.

[43] Kramer J, Özkaya Ö, Kümmerli R (2020). Bacterial siderophores in community and host interactions. Nat Rev Microbiol.

[44] Amanatidou E, Matthews AC, Kuhlicke U, Neu TR, McEvoy JP (2019). Biofilms facilitate cheating and social exploitation of β-lactam resistance in *Escherichia coli*. NPJ Biofilms Microbiomes.

[45] Klümper U, Recker M, Zhang L, Yin X, Zhang T (2019). Selection for antimicrobial resistance is reduced when embedded in a natural microbial community. ISME J.

[46] O’Brien S, Hodgson DJ, Buckling A (2014). Social evolution of toxic metal bioremediation in *Pseudomonas aeruginosa*. Proc Biol Sci.

[47] Velicer GJ, Plucain J (2016). Evolution: bacterial territoriality as a byproduct of kin discriminatory warfare. Curr Biol.

[48] Smith P, Schuster M (2019). Public goods and cheating in microbes. Curr Biol.

[49] Hesse E, O’Brien S, Tromas N, Bayer F, Luján AM (2018). Ecological selection of siderophore-producing microbial taxa in response to heavy metal contamination. Ecol Lett.

[50] Morris JJ, Johnson ZI, Szul MJ, Keller M, Zinser ER (2011). Dependence of the cyanobacterium Prochlorococcus on hydrogen peroxide scavenging microbes for growth at the ocean’s surface. PLoS One.

[51] Cooksey DA (1994). Molecular mechanisms of copper resistance and accumulation in bacteria. FEMS Microbiol Rev.

[52] Cervantes C, Ji G, Ramírez JL, Silver S (1994). Resistance to arsenic compounds in microorganisms. FEMS Microbiol Rev.

[53] O’Brien S, Buckling A (2015). The sociality of bioremediation: Hijacking the social lives of microbial populations to clean up heavy metal contamination. EMBO Rep.

[54] Cazorla FM, Arrebola E, Sesma A, Pérez-García A, Codina JC (2002). Copper resistance in *Pseudomonas syringae* strains isolated from mango is encoded mainly by plasmids. Phytopathology.

[55] Sorg RA, Lin L, van Doorn GS, Sorg M, Olson J (2016). Collective resistance in microbial communities by intracellular antibiotic deactivation. PLoS Biol.

[56] McCutcheon JP, Moran NA (2011). Extreme genome reduction in symbiotic bacteria. Nat Rev Microbiol.

[57] Giovannoni SJ, Cameron Thrash J, Temperton B (2014). Implications of streamlining theory for microbial ecology. ISME J.

[58] Vernyik V, Karcagi I, Tímár E, Nagy I, Györkei Á (2020). Exploring the fitness benefits of genome reduction in *Escherichia coli* by a selection-driven approach. Sci Rep.

[59] D’Souza G, Waschina S, Pande S, Bohl K, Kaleta C (2014). Less is more: selective advantages can explain the prevalent loss of biosynthetic genes in bacteria. Evolution.

[60] Rozen DE, Lenski RE (2000). Long-term experimental evolution in *Escherichia coli*. VIII. Dynamics of a balanced polymorphism. Am Nat.

[61] Adkins-Jablonsky SJ, Clark CM, Papoulis SE, Kuhl MD, Morris JJ (2021). Market forces determine the distribution of a leaky function in a simple microbial community. Proc Natl Acad Sci U S A.

[62] Mas A, Jamshidi S, Lagadeuc Y, Eveillard D, Vandenkoornhuyse P (2016). Beyond the Black Queen hypothesis. ISME J.

[63] Pastore AI, Barabás G, Bimler MD, Mayfield MM, Miller TE (2021). The evolution of niche overlap and competitive differences. Nat Ecol Evol.

[64] Griffin AS, West SA, Buckling A (2004). Cooperation and competition in pathogenic bacteria. Nature.

[65] Foster KR, Bell T (2012). Competition, not cooperation, dominates interactions among culturable microbial species. Curr Biol.

[66] Palmer JD, Foster KR (2022). Bacterial species rarely work together. Science.

[67] Kehe J, Ortiz A, Kulesa A, Gore J, Blainey PC (2021). Positive interactions are common among culturable bacteria. Sci Adv.

[68] Hammarlund SP, Harcombe WR (2019). Refining the stress gradient hypothesis in a microbial community. Proc Natl Acad Sci U S A.

[69] Lawrence D, Fiegna F, Behrends V, Bundy JG, Phillimore AB (2012). Species interactions alter evolutionary responses to a novel environment. PLoS Biol.

[70] Coyte KZ, Schluter J, Foster KR (2015). The ecology of the microbiome: networks, competition, and stability. Science.

[71] Scheuerl T, Hopkins M, Nowell RW, Rivett DW, Barraclough TG (2020). Bacterial adaptation is constrained in complex communities. Nat Commun.

[72] Cairns J, Jokela R, Becks L, Mustonen V, Hiltunen T (2020). Repeatable ecological dynamics govern the response of experimental communities to antibiotic pulse perturbation. Nat Ecol Evol.

[73] Evans R, Beckerman AP, Wright RCT, McQueen-Mason S, Bruce NC (2020). Eco-evolutionary dynamics set the tempo and trajectory of metabolic evolution in multispecies communities. Curr Biol.

[74] Morris JJ, Papoulis SE, Lenski RE (2014). Coexistence of evolving bacteria stabilized by a shared Black Queen function. Evolution.

[75] Perlin MH, Clark DR, McKenzie C, Patel H, Jackson N (2009). Protection of Salmonella by ampicillin-resistant *Escherichia coli* in the presence of otherwise lethal drug concentrations. Proc Biol Sci.

[76] Frost I, Smith WPJ, Mitri S, Millan AS, Davit Y (2018). Cooperation, competition and antibiotic resistance in bacterial colonies. ISME J.

[77] Patel M, Raymond B, Bonsall MB, West SA (2019). Crystal toxins and the volunteer’s dilemma in bacteria. J Evol Biol.

[78] Brockhurst MA, Buckling A, Racey D, Gardner A (2008). Resource supply and the evolution of public-goods cooperation in bacteria. BMC Biol.

[79] Luo H, Csuros M, Hughes AL, Moran MA (2013). Evolution of divergent life history strategies in marine alphaproteobacteria. mBio.

[80] Browne HP, Forster SC, Anonye BO, Kumar N, Neville BA (2016). Culturing of “unculturable” human microbiota reveals novel taxa and extensive sporulation. Nature.

